# Supplementary material for: Development of a cryptocurrency price prediction model: leveraging GRU and LSTM for Bitcoin, Litecoin and Ethereum
Source: PeerJ Comput Sci. 2025 Mar 17;11:e2675. doi: 10.7717/peerj-cs.2675 (PMC11935774; doi:10.7717/peerj-cs.2675)
Supplement: Supplemental Information 1 [file peerj-cs-11-2675-s001.docx]

**Overview**

This paper focuses on predicting cryptocurrency prices (Bitcoin, Ethereum, and Litecoin) using deep learning algorithms, specifically Long Short-Term Memory (LSTM) and Gated Recurrent Unit (GRU). The aim is to improve the accuracy of cryptocurrency price forecasting, making it useful for investors and traders. The implementation involves analyzing historical market data, building predictive models, and comparing their performance using various metrics.

**Data Source**

- **Cryptocurrency Data**: Historical data for Bitcoin (BTC), Ethereum (ETH), and Litecoin (LTC) fetched from [Crypto Data Download](https://www.cryptodatadownload.com/).

**Features of the Code**

1. **Data Processing**:
   - Collected data for BTC, ETH, and LTC between 2017 to 2024.
   - Preprocessing steps include normalization using MinMaxScaler and splitting the dataset into training (80%) and testing (20%) sets.
2. **Model Implementation**:
   - **Long Short-Term Memory (LSTM)**: A type of recurrent neural network designed to retain long-term dependencies in time-series data.
   - **Gated Recurrent Unit (GRU)**: A simplified version of LSTM, designed to perform as well or better with fewer parameters.
   - Both models are tuned and trained on the cryptocurrency datasets.
3. **Model Evaluation**:
   - Performance is evaluated using key metrics: Mean Squared Error (MSE), Root Mean Squared Error (RMSE), Mean Absolute Error (MAE), and Mean Absolute Percentage Error (MAPE).
   - GRU outperforms LSTM in most cases for BTC, ETH, and LTC, with lower error metrics.
4. **Visualization**:
   - Plots for actual vs. predicted prices are generated to visually compare the performance of the models.
   - Training and testing loss graphs help evaluate the model's performance over time.

**Dependencies**

The following Python libraries are required:

- pandas: Data manipulation and analysis.
- numpy: Numerical operations.
- matplotlib and seaborn: Data visualization.
- keras and tensorflow: Deep learning frameworks.
- scikit-learn: Preprocessing and performance metrics.

You can install these dependencies using:

bash

Copy code

pip install pandas numpy matplotlib seaborn keras tensorflow scikit-learn

**How to Run the Notebook**

1. **Run the Jupyter Notebook**:
   - Open **ipynb** file in Jupyter Notebook or JupyterLab.
   - Follow the instructions in the notebook to load the data, preprocess it, train the models (LSTM and GRU), and visualize the results.
2. **Evaluate the Results**:
   - The notebook will output key performance metrics and visualizations, including actual vs. predicted price plots and loss curves.

**Results**

- **LSTM Performance**: While LSTM models show good performance in predicting prices, they tend to smooth out abrupt price swings, which may reduce their accuracy during volatile market conditions.
- **GRU Performance**: GRU models outperform LSTM with lower error rates, making it more suitable for cryptocurrency price predictions.
  - Example Metrics:
    - **GRU RMSE for BTC**: 0.03258
    - **LSTM RMSE for BTC**: 0.06749

**Future Work**

- Explore additional features such as social media sentiment analysis or trading volume to improve prediction accuracy.
- Implement other machine learning models (e.g., ARIMA, XGBoost) for comparison.
- Automate the process by integrating API-based real-time data fetching.
